# Renal Function Improvement by Telbivudine in Liver Transplant Recipients with Chronic Kidney Disease

**DOI:** 10.1155/2017/9324310

**Published:** 2017-08-13

**Authors:** Wei-Chen Lee, Tsung-Han Wu, Yu-Chao Wang, Chih-Hsien Cheng, Chen-Fang Lee, Ting-Jung Wu, Hong-Shiue Chou, Kun-Ming Chan, Ching-Song Lee

**Affiliations:** ^1^Department of Liver and Transplantation Surgery, Chang-Gung Memorial Hospital, Chang-Gung University College of Medicine, Taoyuan, Taiwan; ^2^Department of Hepatology, Chang-Gung Memorial Hospital, Chang-Gung University College of Medicine, Taoyuan, Taiwan

## Abstract

Chronic renal failure is a frequent complication in liver transplantation. Telbivudine, anti-hepatitis B virus (HBV) nucleoside, can improve renal function. It is interesting if using telbivudine for prophylaxis of HBV recurrence has additional value on renal function improvement. 120 liver transplant recipients with lamivudine prophylaxis for HBV recurrence were 1 : 1 randomized into lamivudine-continuous (*n* = 60) and telbivudine-replacement (*n* = 60) groups. Fifty-eight patients in lamivudine-continuous group and 54 in telbivudine-replacement group completed the study. In telbivudine-replacement group, the estimated glomerular filtration rate (eGRF) was improved from 63.0 ± 16.3 ml/min to 72.8 ± 21.1 ml/min at 12 months after telbivudine administration (*p* = 0.003). Stratifying the patients according to renal function staging, the eGRF was improved from 74.7 ± 6.9 ml/min to 84.2 ± 16.6 ml/min (*p* = 0.002) in 32 stage II patients and from 48.2 ± 7.3 ml/min to 59.7 ± 11.8 ml/min in 20 stage III patients after 12 months of telbivudine administration (*p* < 0.001). Eleven (18.3%) patients with telbivudine developed polyneuritis during the trial and post hoc following-up. In conclusion, renal function was improved by telbivudine in liver transplant recipients with long-term chronic kidney disease. However, the high incidence of polyneuritis induced by telbivudine has to be closely monitored. This trial is registered with ClinicalTrials NCT02447705.

## 1. Introduction

Liver transplantation is the most effective treatment for the patients with acute liver failure, end-stage liver diseases, and hepatocellular carcinoma within Milan criteria [1]. Those patients with acute liver failure or end-stage liver diseases are frequently associated with acute/chronic kidney injury. When the model of end-stage liver disease (MELD) was introduced to liver transplantation, allocation priority of deceased liver allografts was determined by MELD scores [2, 3]. As serum creatinine is a component of MELD calculating formula, most of the liver transplant recipients have renal injury when liver allografts are allocated and transplantations are performed.

After liver transplantation, immunosuppression has to be maintained by lifelong immunosuppressive agents. Calcineurin inhibitors (CNI), tacrolimus and cyclosporine, are employed as the key components of various immunosuppressive regimens [4, 5]. It is well known that nephrotoxicity is one of the adverse effects of tacrolimus and cyclosporine [5]. The administration of tacrolimus or cyclosporine may further deteriorate the already damaged renal function [6]. Ojo et al. reported that chronic renal failure developed in 16.5% of nonrenal organ transplant recipients in a median follow-up of 3 years and the cumulative incidence of chronic renal failure in liver transplant recipients was as high as 18.1% in 5 years [7]. Because chronic renal failure after transplantation is associated with high mortality, improvement of renal function in transplant recipient is paramount.

Antiviral agents with short- or long-term anti-hepatitis B immunoglobulin are essential to prevent hepatitis B recurrence after liver transplantation [8]. Lamivudine (LAM), a nucleoside analogue, is the first available oral anti-hepatitis B virus (HBV) agent and is still a popular agent to prevent HBV recurrence in liver transplantation [9, 10]. Although LAM is recognized nontoxic to renal function, renal function declines in a long-term administration [11]. Telbivudine (LdT) is another approved anti-HBV nucleoside. Recently, studies on chronic hepatitis B patients showed that LdT could improve eGFR during chronic hepatitis B treatment [11, 12]. In liver transplantation, whether LdT can be applied for prophylaxis of HBV recurrence and improves long-term injured renal function simultaneously is still unclear. In this study, liver transplant recipients with long-term LAM for prophylaxis of HBV recurrence was randomized into two groups to see whether renal function could be improved by switching LAM to LdT.

## 2. Materials and Methods

### 2.1. Eligible Patients and Inclusion Criteria

The recipients of liver transplantation with LAM for prophylaxis of HBV recurrence were eligible patients. The inclusion criteria for patient enrollment included age ≥ 18 years, stable liver function for more than 6 months, renal function in stages 2–4 [13], and signing informed consent. We hypothesized that renal function would be improved in ≥20% of the patients with LdT instead of LAM. Therefore, the number of patients enrolled in each group would be 45 at least at a power of 0.80 and *α* = 0.05. The patients were enrolled by transplant surgeons who cared for the patients in clinic.

### 2.2. Study Protocol

The enrolled patients were randomized 1 : 1 into two groups: LAM-continuous (LAM-C) group and LdT-replacement (LdT-R) group by a set of computer-generated random numbers. The participants were assigned to each group according to random numbers by study-nursing staff. In LAM-C group, the prophylaxis of HBV recurrence was not changed and the dose of LAM was kept one tablet a day (100 mg/day). In LdT-R group, the prophylaxis of HBV recurrence was shifted from LAM to LdT and the dose of LdT was one tablet a day (600 mg/day). This study was carried on for one year and renal function was documented. This study protocol conformed to the ethical guidelines of the 1975 Declaration of Helsinki and was approved by institutional review board of Chang-Gung Memorial Hospital (CGMH-IRB-101-3476A3) in April, 2013, and registered in ClinicalTrials (NCT02447705) in June, 2013.

### 2.3. Patients Withdrawing

The patients having allergy to LdT would be withdrawn from the study. During the study period, the patients would be withdrawn from this study when they suffered from cancer or severe infection with medications which might interact with CNI and interfere with renal or liver function. The patients having severe adverse effects of LdT would also be withdrawn from this study.

### 2.4. Immunosuppression

All patients enrolled in this study were in stable liver function. Immunosuppressive regimen consisted of tacrolimus/cyclosporine with mycophenolate mofetil (MMF). All patients were all steroid-free when they were enrolled in the study.

### 2.5. Anti-HBV Immunoglobulin for Prophylaxis of Hepatitis B Recurrence

All the patients in this study were positive for HBV surface antigen before transplantation. During operation, 10000 U of anti-HBV immunoglobulin (HBIg) was given intravenously at anhepatic phase. After transplantation, 2000 U of HBIg was administered intravenously every day from postoperative day (POD) 1 to 7. LAM was taken and continued from POD 1.

### 2.6. Follow-Up

All the patients were followed up every two months. Blood samples were taken for measurements of liver function, renal function, presence of surface antigen of hepatitis B (HBs Ag), and trough levels of tacrolimus or cyclosporine. Renal function was presented by eGRF which was calculated by Modification of Diet in Renal Disease (MDRD) formula [14].

### 2.7. Biostatistics

The paired and unpaired Student's *t*-tests were used to analyze continuous variables. Categorical variables were analyzed by either the Chi-square test or Fisher's exact test. All multiple pairwise comparisons were done using comparison ANOVA with the Holm-Sidak correction. All statistical analyses were performed with SigmaPlot 12.3 software for Windows (Systat Software, Inc., San Jose, CA, USA).* p* < 0.05 was considered statistically significant.

## 3. Results

### 3.1. Patients

**Figure 1:**
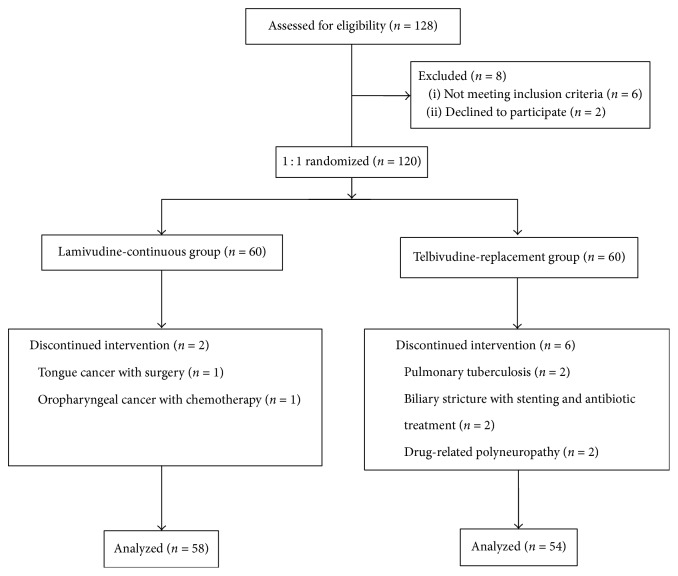
The flow diagram of 120 patients enrolled in this study. Each arm consisted of 60 patients. Two patients in LAM-C arm and 6 patients in LdT-R arm were withdrawn. Finally, 58 patients in LAM-C arm and 54 patients in LdT-R arm completed the study.

Totally, 120 patients were enrolled in this study and randomized 1 : 1 into LAM-C and LdT-R arms from June, 2013 (Figure 1). Each arm consisted of 60 patients. Two patients in LAM-C arm were withdrawn from the study because they suffered from tongue/oropharyngeal cancer and received chemotherapy. Six patients in LdT-R arm were withdrawn because two patients had pulmonary tuberculosis, two had biliary tree infection with antibiotics treatment, and the other two had peripheral neuropathy related to LdT. Finally, 58 patients in LAM-C arm and 54 patients in LdT-R arm completed the study. The ages between LAM-C arm and LdT-R arm were not different (58.3 ± 7.4 versus 58.2 ± 8.1 years old,* p* = 0.903). The median (interquartile) posttransplant time for the patients in LdT-R arm was 47.0 (28.1 to 74.6) months which was not different from 59.4 (36.0 to 89.4) months for the patients in LAM-C arm (*p* = 0.182). The renal function staging was compatible for the patients in both arms and the liver function for most of the patients was within normal limits (Table 1).

### 3.2. Immunosuppression

**Figure 2:**
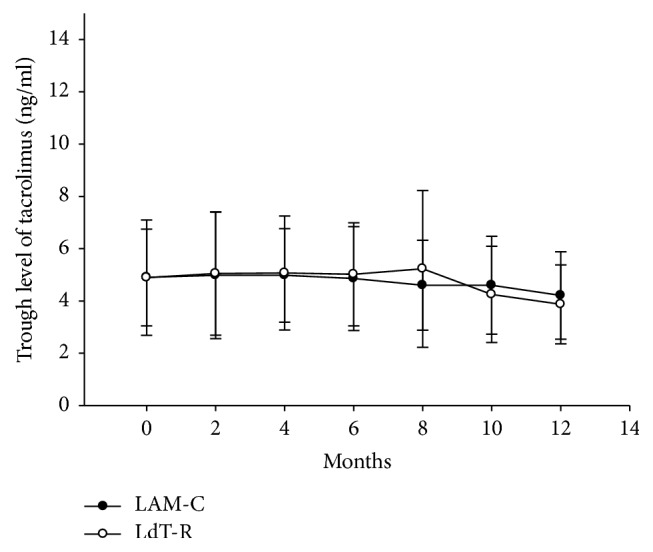
The trough levels of tacrolimus during the study. The trough blood levels of tacrolimus were 4.90 ± 1.85 in LAM-C group and 4.89 ± 2.22 ng/ml in LdT-R group when the patients were enrolled in the study (*p* = 0.588). During the study, trough levels of tacrolimus were slightly declined. At the end of this study, the trough blood levels of tacrolimus were 4.21 ± 1.67 ng/ml in LAM-C group and 3.87 ± 1.51 ng/ml in LdT-R group (*p* = 0.317).

In LAM-C arm, 57 patients took tacrolimus and 1 patient took cyclosporine. In LdT-R arm, 53 patients took tacrolimus and 1 patient took cyclosporine. All patients had MMF (500–1000 mg/day). The doses of tacrolimus between two arms were not different when the patients were enrolled in the study (3.13 ± 1.69 versus 3.26 ± 1.41 mg/day,* p* = 0.419). The trough blood levels of tacrolimus between two arms were not different, either (4.90 ± 1.85 versus 4.89 ± 2.22 ng/ml,* p* = 0.588). During the study, trough levels of tacrolimus were slightly declined. At the end of this study, the trough blood levels of tacrolimus between two arms were not different, either (4.21 ± 1.67 versus 3.87 ± 1.51 ng/ml,* p* = 0.317) (Figure 2).

### 3.3. Hepatitis B Recurrence

When the patients were enrolled in the study, two patients in LAM-C arm were positive for HBs Ag, but their DNA of HBV was negative. All patients in LdT-R arm were negative for HBs Ag. At the end of the study, the two patients in LAM-C arm remained positive for HBs Ag. No other patients in both arms had HBV recurrence.

### 3.4. Improvement in Renal Function

**Figure 3:**
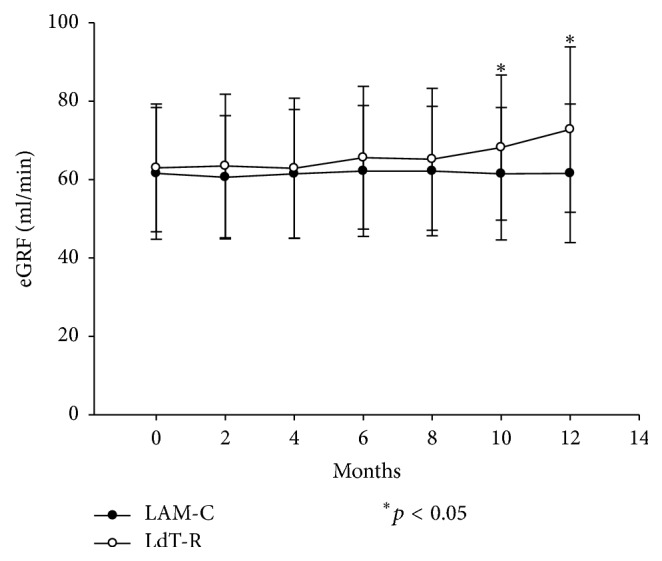
The eGRF for the patients in the two arms. At the beginning, the baseline eGRF of LdT-R arm patients was 63.0 ± 16.3 ml/min which was not different from 61.6 ± 16.8 ml/min of LAM-C arm patients (*p* = 0.645). The renal function was improved for the patients in LdT-R arm from 10 months after LdT administration and reached 72.8 ± 21.1 ml/min at 12 months (*p* = 0.003).

The eGRF (ml/min/1.73 m^2^) was calculated by MDRD formula when the patients were enrolled in the study. Then, the eGRF was calculated every 2 months until the end of the study. At the beginning, the baseline eGRF of the patients in LdT-R arm was 63.0 ± 16.3 ml/min which was not different from 61.6 ± 16.8 ml/min in the patients in LAM-C arm (*p* = 0.645). During the study period, eGRF of the patients in LAM-C arm was almost the same until the end of the study (from 61.6 ± 16.8 to 61.6 ± 17.7 ml/min, *p* = 0.686). However, the renal function was improved for the patients in LdT-R arm from 10 months after LdT administration and eGFR was improved to 68.2 ± 18.5 ml/min at 10 months (*p* = 0.026) and 72.8 ± 21.1 ml/min at 12 months (*p* = 0.003). The 95 percent two-tailed confidence interval for difference of means at 12 months was 3.966 to 18.528 ml/min of eGFR (Figure 3).

### 3.5. Renal Function Improvement according to Staging

In LAM-C arm, 33 patients were in stage II renal function and their eGRF was from baseline 74.0 ± 7.5 ml/min to 73.1 ± 9.2 ml/min at the end of one year (*p* = 0.471); 23 patients were in stage III renal function and their eGRF was from baseline 47.0 ± 8.8 ml/min to 49.0 ± 11.3 ml/min at the end of one year (*p* = 0.058). Therefore, the renal function of the patients in LAM-C arm was not changed no matter their renal function was in stage II or III before enrollment in this study.

**Figure 4:**
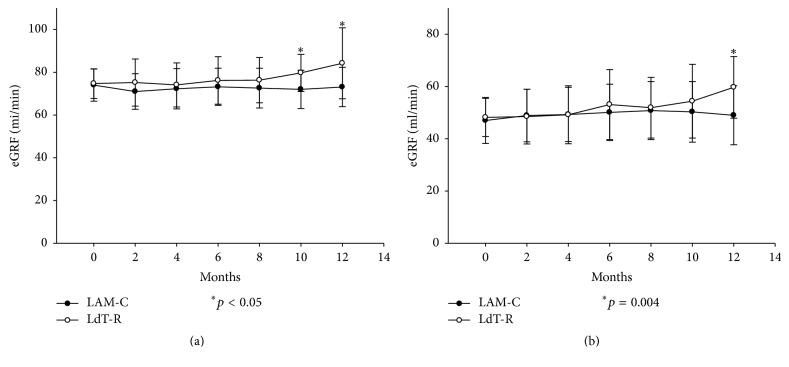
The eGRF for the patients with renal function in stage II or III. (a) For the 32 patients in stage II in LdT-R arm, eGRF was improved from baseline 74.7 ± 6.9 ml/min to 79.7 ± 8.7 ml/min at 10 months of LdT administration (*p* = 0.007) and 84.2 ± 16.6 ml/min at 12 months of LdT administration (*p* = 0.002). Compared to LAM-C arm, eGFR became different from 10 months after LdT administration. (b) For the 20 patients in stage III, eGRF was improved from baseline 48.2 ± 7.3 ml/min to 54.4 ± 14.1 ml/min at 10 months of LdT administration (*p* = 0.002) and 59.7 ± 11.8 ml/min at 12 months of LdT administration (*p* < 0.001). Compared to LAM-C arm, eGFR became improved at 12 months after LdT administration.

In LdT-R arm, 32 patients were in stage II, 20 patients in stage III, and 2 patients in stage IV before being enrolled in the study. For the 32 patients in stage II, eGRF was improved from baseline 74.7 ± 6.9 ml/min to 79.7 ± 8.7 ml/min at 10 months (*p* = 0.007) and 84.2 ± 16.6 ml/min at 12 months of LdT administration (*p* = 0.002). Nine (28.1%) patients' renal function was improved to stage I. The 95% confidence interval for improvement of eGFR was from 3.6 to 15.3 ml/min at 12 months after LdT-replacement (Figure 4(a)). For the 20 patients in stage III, eGRF was improved from baseline 48.2 ± 7.3 ml/min to 54.4 ± 14.1 ml/min at 10 months of LdT administration (*p* = 0.002) and 59.7 ± 11.8 ml/min at 12 months of LdT administration (*p* < 0.001). Eleven (55%) patients' renal function was improved from stage III to stage II. The 95% confidence interval for difference of eGFR was from 7.1 to 15.8 ml/min at 12 months after LdT-replacement (Figure 4(b)). For the 2 patients in stage IV renal function, eGRF was not improved after LdT administration.

### 3.6. The Population with Renal Function Improvement in Both Arms

**Figure 5:**
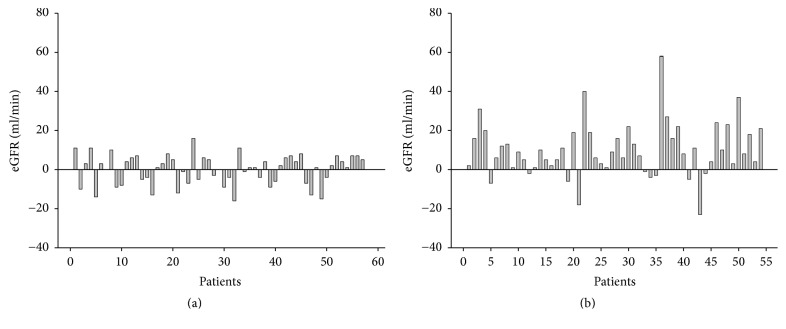
The change of eGFR for an individual patient between initiation and end of the study. (a) In LAM-C arm, 32 (55.2%) patients had better eGFR, 23 (39.6%) patients had worse eGFR, and 3 (5.2%) patients did not have any change. Among them, 22 (37.9%) patients had their eGFR improvement ≥4 ml/min. (b) In LdT-R arm, 44 (81.5%) patients had better eGFR and 10 (18.5%) patients had worse eGFR. Among them, 37 (68.5%) patients had their eGFR improvement ≥4 ml/min.

In LAM-C arm, 32 (55.2%) patients had better eGFR, 23 (39.6%) patients had worse eGFR, and 3 (5.2%) patients did not have any change. In LdT-R arm, 44 (81.5%) patients had better eGFR and 10 (18.5%) patients had worse eGFR. The 95 percent two-tailed confidence interval for renal function improvement of means between LAM-C and LdT-R arms at 12 months was 3.966 to 18.528 ml/min of eGFR. Therefore, we defined that the renal function was improved if increase of eGFR was ≥4 ml/min at the end of this study. Twenty-two (37.9%) patients in LAM-C arm (Figure 5(a)) and 37 (68.5%) patients in LdT-R arm improved their renal function at the end of this study (Figure 5(b), *p* = 0.002).

### 3.7. The Relationship between Alternations of Tacrolimus Trough Levels and eGFR

**Figure 6:**
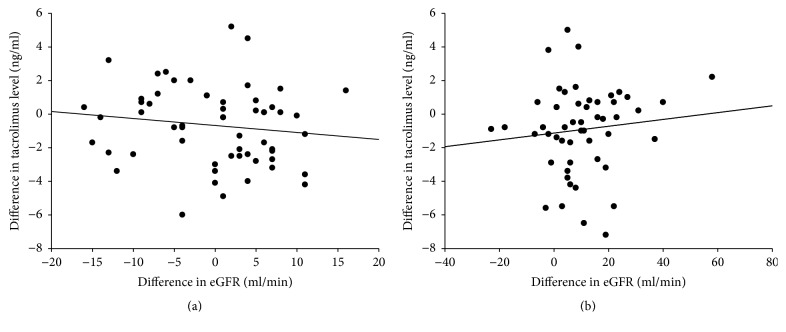
The relationship between alternation of tacrolimus and eGFR at the end of the study. (a) In LAM-C arm, the alternation of eGFR was not correlated to the alternation of tacrolimus trough levels (*R*^2^ = 0.0183). (b) In LdT-R arm, the alternation of eGFR was not correlated to the alternation of tacrolimus trough levels, either (*R*^2^ = 0.0119).

In this study, the trough levels of tacrolimus at the end of the study were lower than those when the patients were enrolled in both arms. To determine whether the renal function improvement was related to decease of tacrolimus trough levels, the relationship between the alternations of eGFR and tacrolimus trough levels was examined. The results showed that the increase of eGFR was not correlated to the decrease of tacrolimus trough level in both LAM-C arm (Figure 6(a)) and LdT-R arm (Figure 6(b)).

### 3.8. Adverse Effects of LdT

**Figure 7:**
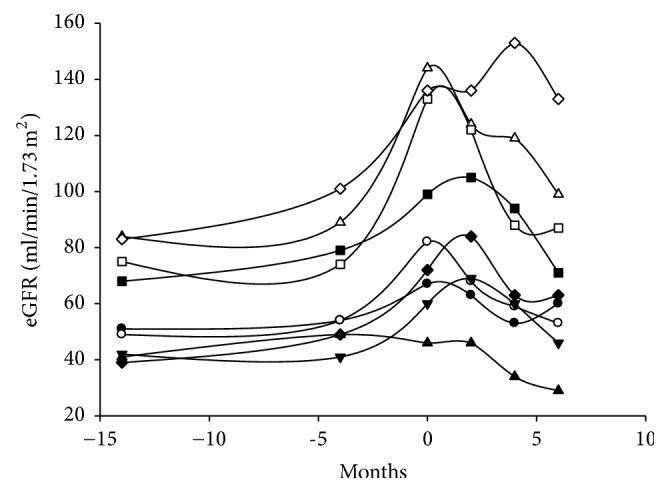
The eGFR for the patients in LdT arm and switched back to LAM. During post hoc following-up, 9 patients had polyneuropathy and were switched back to LAM. While LdT was switched back to LAM, eGFR was kept in improved renal function in 5 (55.6%) patients and returned to original levels in 4 (44.4%) patients.

During the study period, 2 patients complained of numbness over toes and felt weakness of the thighs when they were going to stand up. Neurological examinations were performed. Somatosensory evoked potential showed sensory conducted defect in bilateral low limbs and muscular electronic potential was normal. Polyneuritis was diagnosed and the patients were withdrawn from the study. During post hoc following-up, another 9 patients had similar symptoms and were switched back to LAM. While LdT was switched back to LAM, the eGFR was kept in improved renal function in 5 (55.6%) patients and returned to original levels in 4 (44.4%) patients (Figure 7). Finally, the incidence of polyneuritis induced by LdT was 18.3% (*n* = 11/60). The median (interquartile) time of polyneuritis attack was 392 (376–450) days with a range from 203 to 483 after LdT administration. After LdT was discontinued, the weakness of the thighs was recovered. However, two patients complained of persistent numbness over the tips of toes.

### 3.9. Post Hoc Following-Up

The renal function was followed up continuously after the end of this clinical trial. In LAM-C group, eGFR was 61.6 ± 19.5 ml/min at 18 months and 61.5 ± 20.4 ml/min at 24 months which were not different from 61.6 ± 17.7 ml/min at 12 months (*p* = 0.945). In LdT-R group, eGFR was 72.8 ± 21.2 ml/min at 12 months and increased to 80.1 ± 27.6 ml/min at 18 months (*p* = 0.002) and 79.0 ± 32.5 ml/min at 24 months (*p* < 0.001). This result implied that eGRF was continuously improved until 18 months after medication shifting.

## 4. Discussion

Chronic kidney dysfunction is recognized as a frequent complication in organ transplantation because of long-term use of calcineurin inhibitors for maintaining immunosuppression. Before MELD era for liver allograft allocation, Ojo et al. reported that the cumulative incidence of chronic renal failure increased over time and 16.5% of nonrenal solid organ transplant recipients developed chronic renal failure after a median 3-year following-up [7]. In liver transplantation, Pawarode et al. reported that 35% of their patients developed permanent renal dysfunction and 7% of the patients developed severe renal failure [14]. It was also well known that renal function was an important factor contributing to transplant recipients' survival and the survival would be shortened if renal function was impaired [14, 15]. In MELD era, liver allograft allocation or priority of liver transplantation is according to the MELD scores. As serum creatinine level is one of the components in calculation, liver transplantation recipients frequently have renal function impairment before transplantation. After transplantation, CNI is the main immunosuppressive agent to achieve immunosuppression; however, its adverse effect on renal function may further damage the preexisted renal dysfunction [16, 17]. There is no doubt that to keep kidney in good function is more crucial than before for liver transplant recipients. Nevertheless, it is hard to find an available treatment to reverse the impaired renal function until now.

HBV-related liver disease is one of the major indications of liver transplantation, particularly in Asian countries. Chronic HBV with decompensated liver function may impair renal function [18]. After transplantation, prevention of HBV recurrence is essential [8, 19]. No matter long- or short-term anti-HBV immunoglobulin is applied, oral antiviral agents are administered for lifelong and renal function impairment is concerned. Lamivudine is the oldest one and safe for long-term use in liver transplantation [9, 10]. Although LAM was not toxic to renal function, renal function declined after a long-term use [20]. Telbivudine was reported to improve renal function recently [11, 20]. Therefore, liver transplantation recipients for HBV-related diseases with LdT for prophylaxis of HBV recurrence might prevent HBV recurrence and add the value of improving renal function [21–23]. Currently, the information from a randomized trial of LdT administration to improve renal function in liver transplantation was limited. This study would be meaningful to examine whether renal function could be improved by LdT when nephrotoxic calcineurin inhibitors were applied simultaneously in the long term for prevention of acute rejection in transplantation recipients.

According to the results, LdT could improve renal function in liver transplantation recipients. In this study, the patients with switching from LAM to LdT improved their renal function. The improvement of renal function was noticed 10 months after LdT switching and continued to 18 months. In the literature, LdT showed its ability to protect or improve renal function at 6 months of LdT administration although the mechanisms were still needed to be determined [24–26]. Obviously, improvement of renal function in transplantation recipients was slower than that in de novo HBV patients. However, LdT definitely could improve renal function in liver transplantation recipients even CNI was applied to maintain immunosuppression for several years.

Renal function could be improved by LdT in liver transplant recipients with mild to moderate renal function impairment. In this study, the patients were substratified according to the stages of renal function impairment. The eGRF was improved by 3.6 to 15.3 ml/min in stage II patients and 7.1 to 15.8 ml/min in stage III patients. Renal function was not improved in stage IV patients although only 2 patients in stage IV were included in this study. In the literature, there is no clear data to show what stage of chronic kidney diseases could be reversed by LdT. Turan et al. roughly described that 89% of their patients in stage III and IV CKD improved which made 70% of the patients in stage II at 48 weeks of LdT administration [23]. In our study, 68.5% of the patients in LdT-R arm improved their renal function, but the data only showed that renal function could be improved by LdT if renal function impairment was in stage II or III. When renal function was already deteriorated to stage IV, the improvement of renal function by LdT was not seen. Another important finding was the improved renal function which persisted in only 55.6% of the patients if LdT was discontinued.

Extrahepatic symptoms are additional concerned issues when anti-HBV nucleos(t)ides were applied to treat chronic HBV. In the study period, two patients had peripheral neuropathy and were withdrawn from the study. Another 9 patients had similar symptoms in post hoc following-up. Therefore, the total incidence of peripheral neuropathy was 18.3% in this study. The median time of peripheral neuropathy development was 392 days. In GLOBE study, peripheral neuropathy only developed in 1.2% of the patients in their 4-year course of the study [12]. Clearly, the incidence of peripheral neuropathy in liver transplantation recipients was much higher than that in de novo HBV patients. Turan et al. also mentioned that the incidence of polyneuropathy and myopathy was high in their liver transplantation recipients [23]. Considerably, this high incidence of peripheral neuropathy might be related to superimpose to CNI which was neurotoxic. However, the exact mechanism of peripheral neuropathy is needed to be determined. Fortunately, the neuropathy was reversible when LdT was discontinued. Therefore, when LdT was applied to prevent HBV recurrence in liver transplantation recipients, peripheral neuropathy might be developed late and should be kept in mind. If peripheral neuropathy was developed, LdT should be discontinued immediately and switched to other anti-HBV nucleos(t)ides.

In conclusion, switching LAM to LdT could improve renal function in stage II and III patients. However, the incidence of reversible peripheral neuropathy in transplantation recipients was high. We should be careful to monitor peripheral neuropathy when LdT was applied to improve or protect renal function in liver transplantation recipients.

## Figures and Tables

**Table 1:** The characteristics of 58 patients in LAM-C arm and 54 patients in LdT-R arm.

	LAM-C	LdT-R	*p*
Age (years)	59 (53–63) [39–74]	59 (51–64) [40–75]	0.903
(Q1, Q3) [range]			
Gender (male, %)	54 (93.1%)	44 (81.5%)	0.116
Allograft			
Deceased	13 (22.4%)	22 (40.7%)	0.059
Living	45 (77.6%)	32 (59.3%)	
Time after transplantation (months)	59.4 (36.0–89.4) [67–165.2]	47.0 (28.1–74.6) [8.1–186.8]	0.182
AST (u/L)	23.5 (19–33.5) [2–106]	20.5 (18–25.5) [13–111]	0.075
ALT (u/L)	22.5 (14–38) [7–105]	18 (12–27) [9–70]	0.035
Tacrolimus			
Doses (mg)	3.13 ± 1.69	3.26 ± 1.41	0.419
Trough level(ng/ml)	4.90 ± 1.85	4.89 ± 2.22	0.588
Renal function			0.960
Stage II	33 (56.9%)	32 (59.3%)	
Stage III	23 (39.7%)	20 (37.0%)	
Stage IV	2 (3.4%)	2 (3.7%)	

(Q1, Q3): interquartile.

## Abbreviations

HBV: Hepatitis B virus
eGFR: Estimated glomerular filtration rates
MELD: The model of end-stage liver disease
CNI: Calcineurin inhibitor
LAM: Lamivudine
LdT: Telbivudine
LAM-C: LAM-continuous
LdT-R: LdT-replacement
MDRD: Modification of diet in renal disease.
